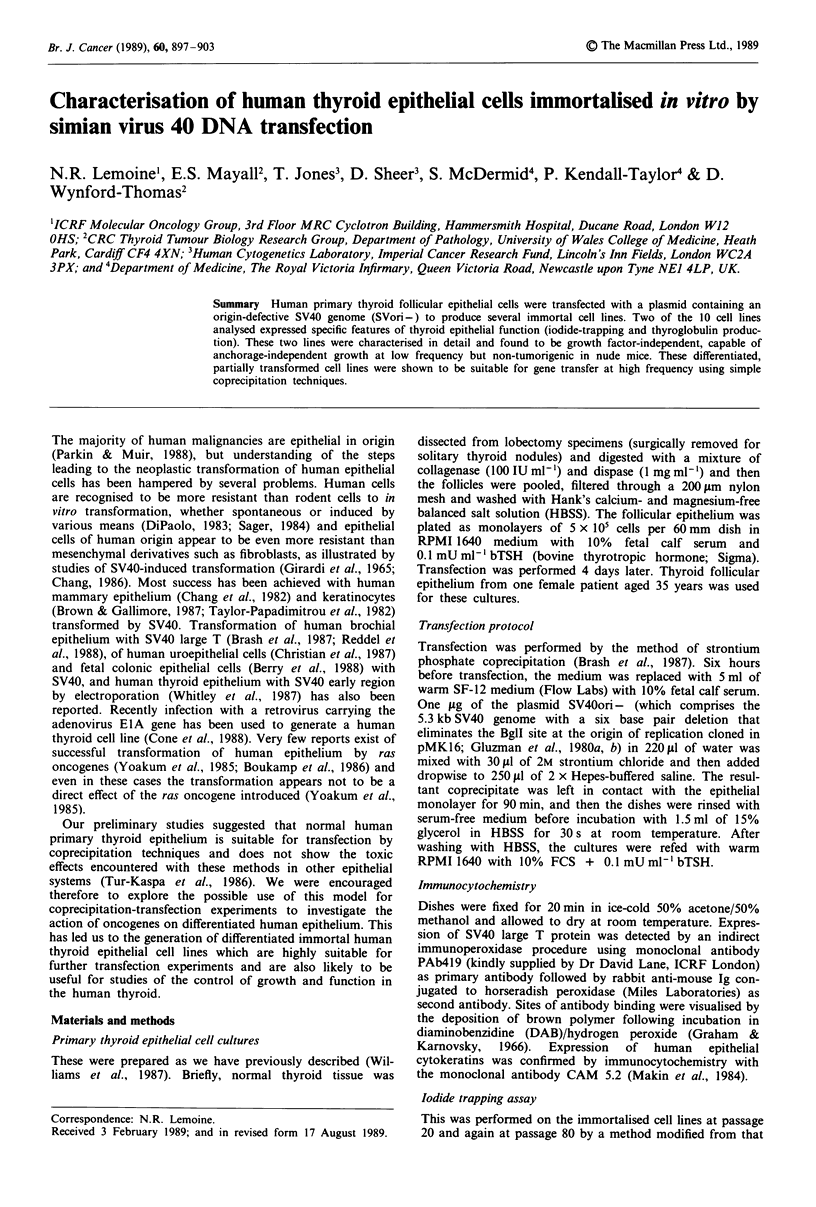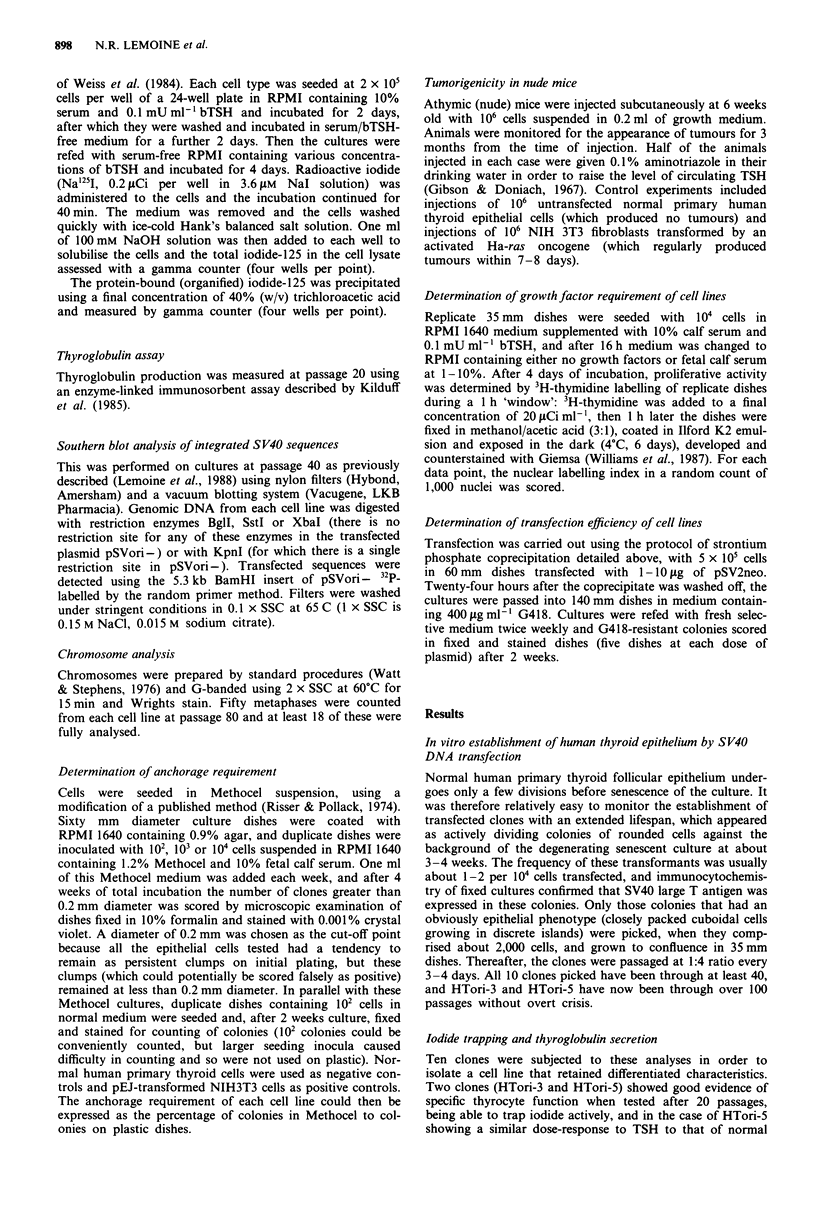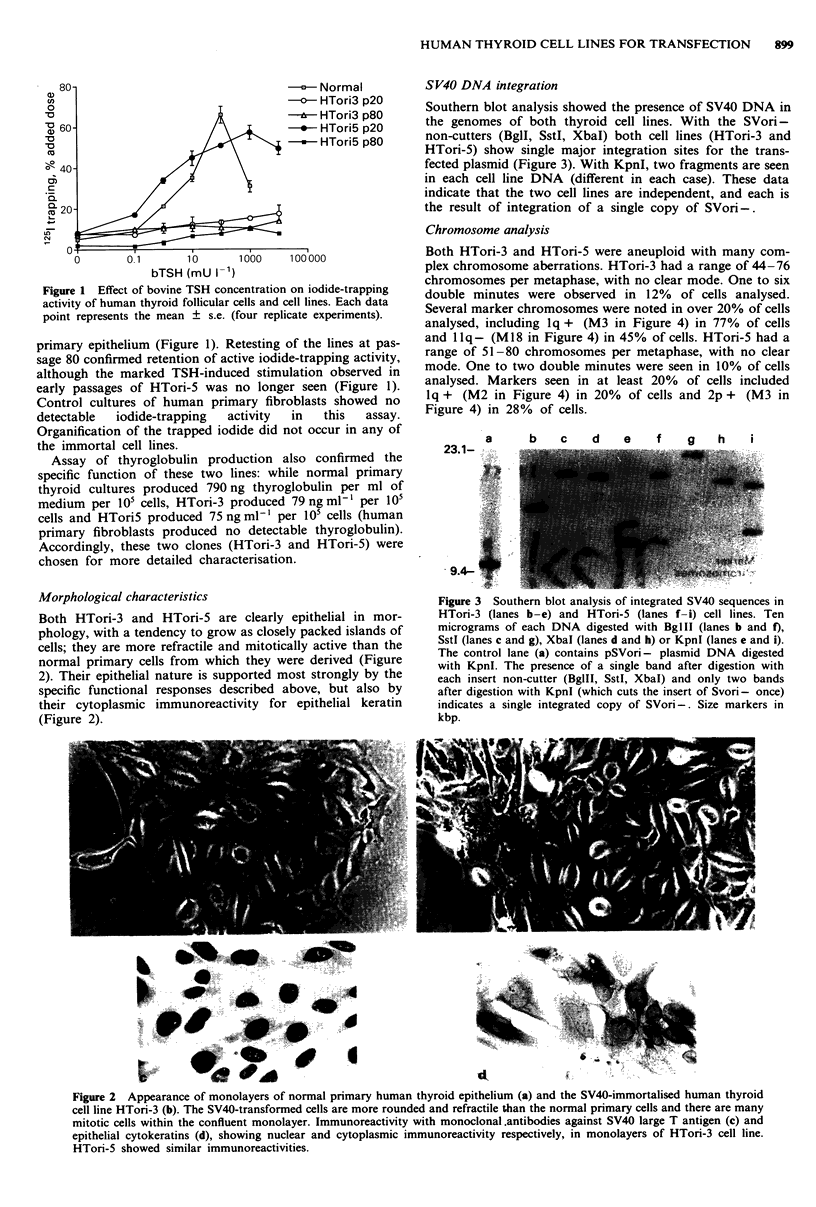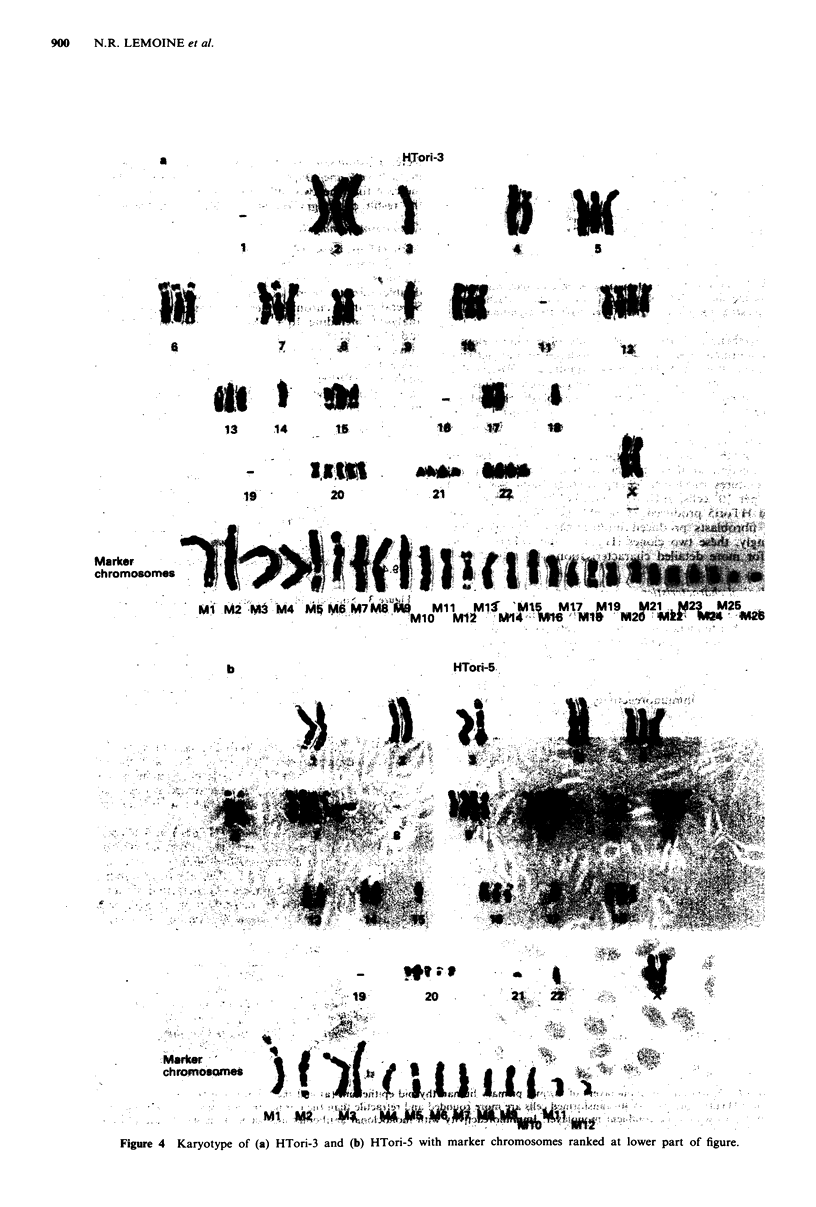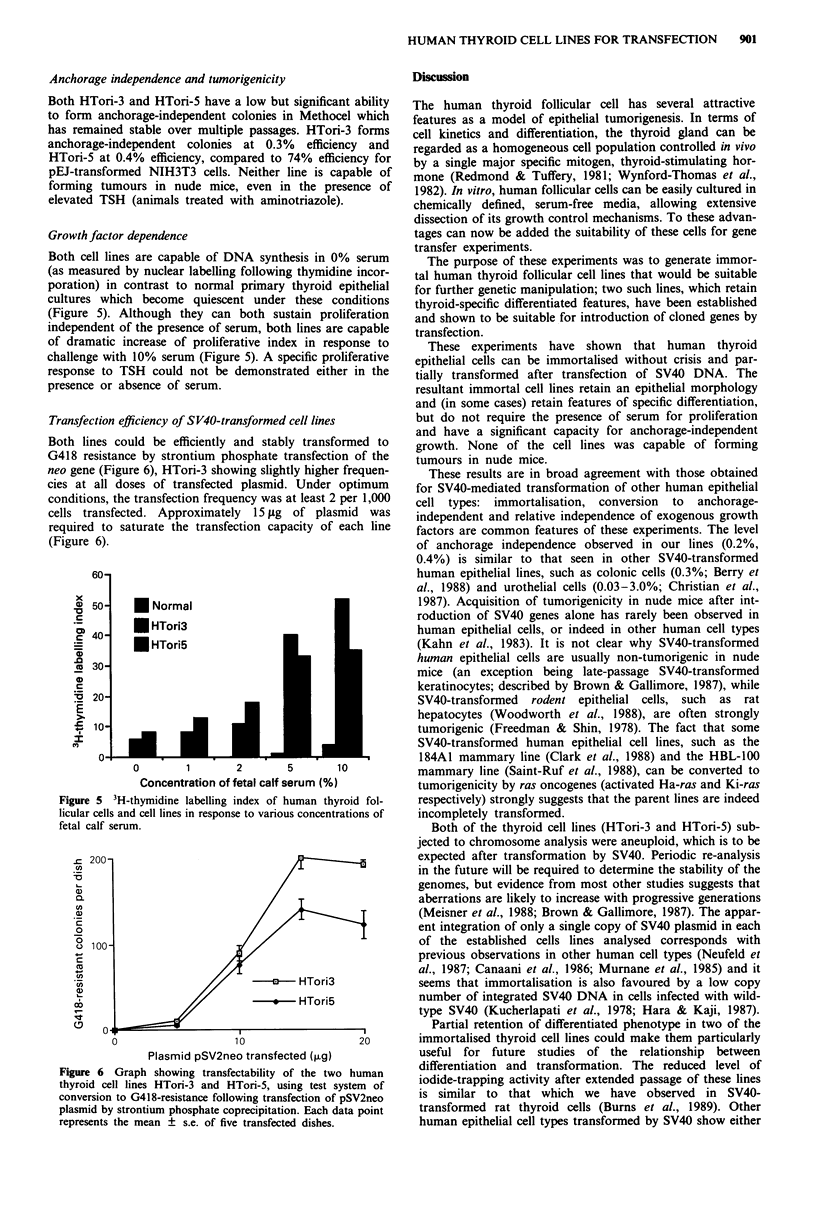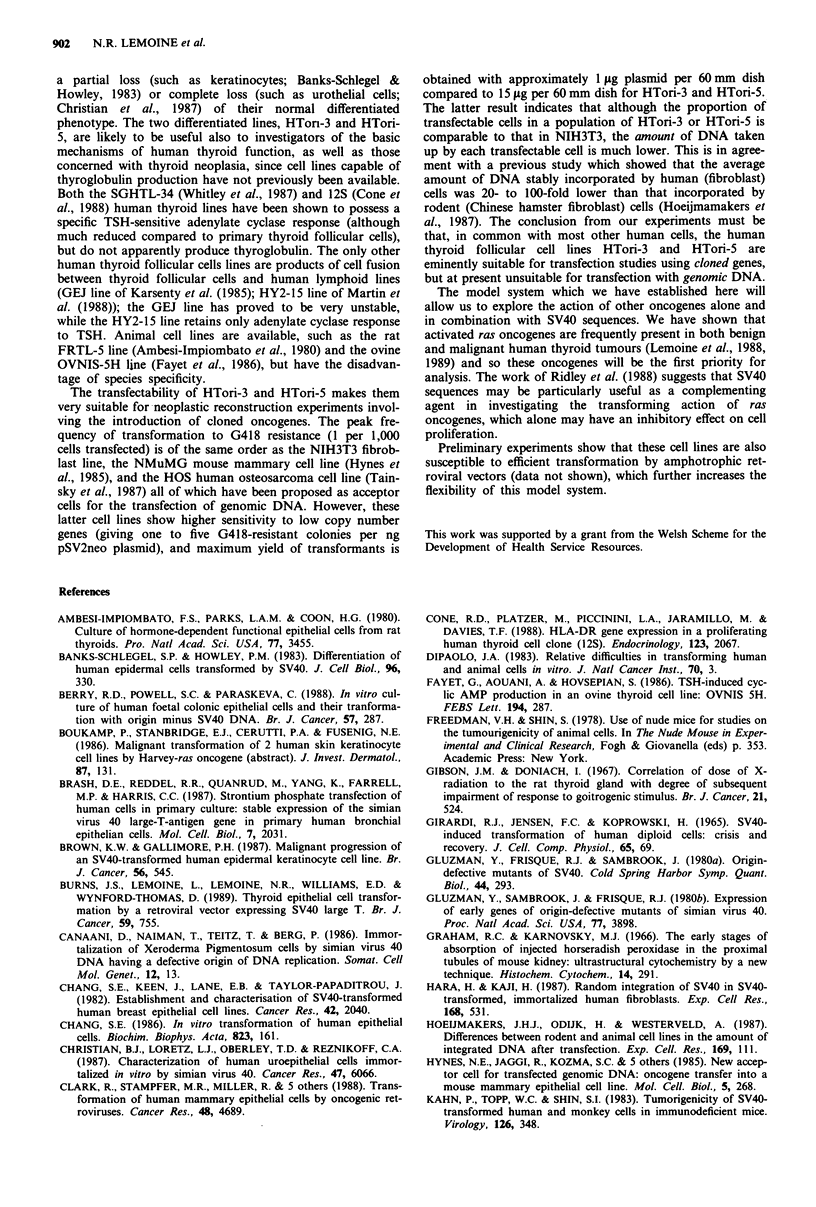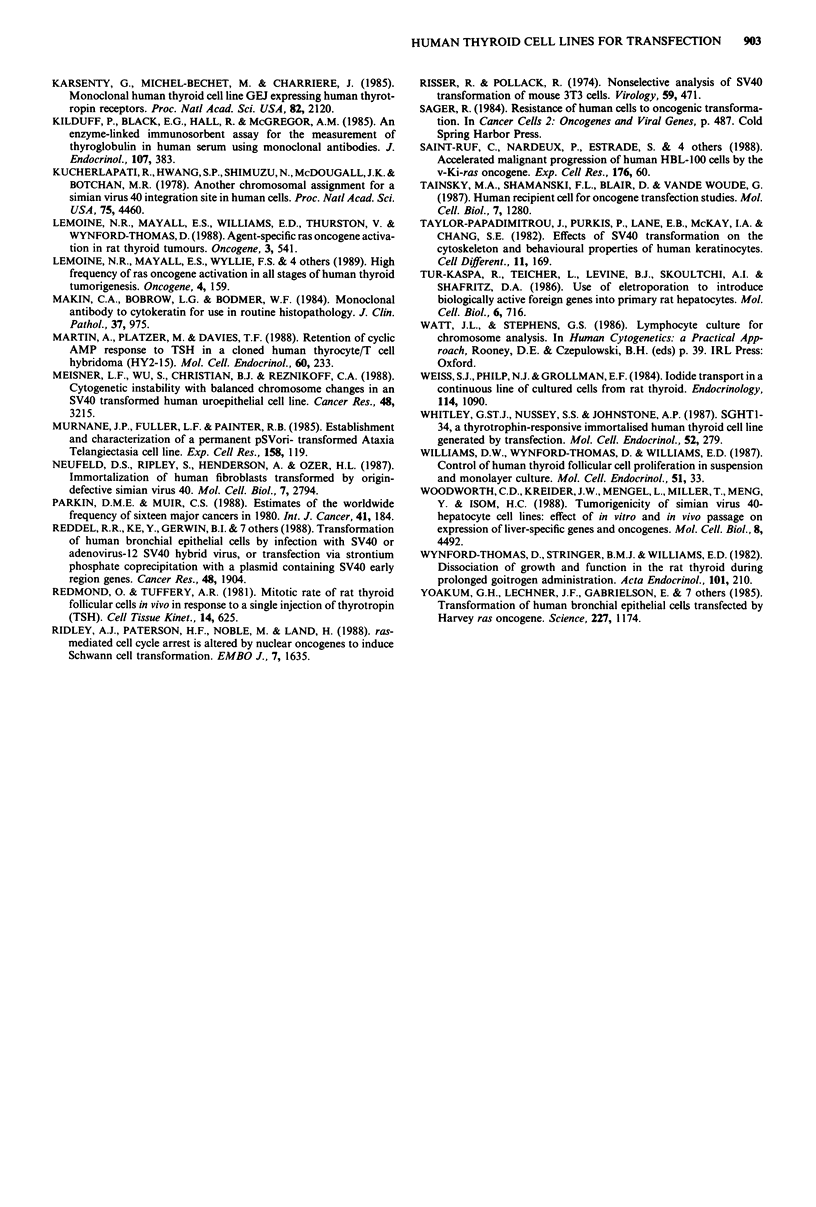# Characterisation of human thyroid epithelial cells immortalised in vitro by simian virus 40 DNA transfection.

**DOI:** 10.1038/bjc.1989.387

**Published:** 1989-12

**Authors:** N. R. Lemoine, E. S. Mayall, T. Jones, D. Sheer, S. McDermid, P. Kendall-Taylor, D. Wynford-Thomas

**Affiliations:** ICRF Molecular Oncology Group, Hammersmith Hospital, London, UK.

## Abstract

**Images:**


Br. J. Cancer (1989), 60, 897-903 © The Macmillan Press Ltd., 1989

### Characterisation of human thyroid epithelial cells immortalised *in vitro* by simian virus 40 DNA transfection

N.R. Lemoine[1], E.S. Mayall[2], T. Jones[3], D. Sheer[3], S. McDermid[4], P. Kendall-Taylor[4] & D. Wynford-Thomas[2]

[1]*ICRF Molecular Oncology Group, 3rd Floor MRC Cyclotron Building, Hammersmith Hospital, Ducane Road, London W12 0HS;* [2]*CRC Thyroid Tumour Biology Research Group, Department of Pathology, University of Wales College of Medicine, Heath Park, Cardiff CF4 4XN;* [3]*Human Cytogenetics Laboratory, Imperial Cancer Research Fund, Lincoln's Inn Fields, London WC2A 3PX; and* [4]*Department of Medicine, The Royal Victoria Infirmary, Queen Victoria Road, Newcastle upon Tyne NE1 4LP, UK.*

**Summary** Human primary thyroid follicular epithelial cells were transfected with a plasmid containing an origin-defective SV40 genome (SVori−) to produce several immortal cell lines. Two of the 10 cell lines analysed expressed specific features of thyroid epithelial function (iodide-trapping and thyroglobulin production). These two lines were characterised in detail and found to be growth factor-independent, capable of anchorage-independent growth at low frequency but non-tumorigenic in nude mice. These differentiated, partially transformed cell lines were shown to be suitable for gene transfer at high frequency using simple coprecipitation techniques.

The majority of human malignancies are epithelial in origin (Parkin & Muir, 1988), but understanding of the steps leading to the neoplastic transformation of human epithelial cells has been hampered by several problems. Human cells are recognised to be more resistant than rodent cells to *in vitro* transformation, whether spontaneous or induced by various means (DiPaolo, 1983; Sager, 1984) and epithelial cells of human origin appear to be even more resistant than mesenchymal derivatives such as fibroblasts, as illustrated by studies of SV40-induced transformation (Girardi *et al*., 1965; Chang, 1986). Most success has been achieved with human mammary epithelium (Chang *et al*., 1982) and keratinocytes (Brown & Gallimore, 1987; Taylor-Papadimitrou *et al*., 1982) transformed by SV40. Transformation of human brochial epithelium with SV40 large T (Brash *et al*., 1987; Reddel *et al*., 1988), of human uroepithelial cells (Christian *et al*., 1987) and fetal colonic epithelial cells (Berry *et al*., 1988) with SV40, and human thyroid epithelium with SV40 early region by electroporation (Whitley *et al*., 1987) has also been reported. Recently infection with a retrovirus carrying the adenovirus E1A gene has been used to generate a human thyroid cell line (Cone *et al*., 1988). Very few reports exist of successful transformation of human epithelium by *ras* oncogenes (Yoakum *et al*., 1985; Boukamp *et al*., 1986) and even in these cases the transformation appears not to be a direct effect of the *ras* oncogene introduced (Yoakum *et al*., 1985).

Our preliminary studies suggested that normal human primary thyroid epithelium is suitable for transfection by coprecipitation techniques and does not show the toxic effects encountered with these methods in other epithelial systems (Tur-Kaspa *et al*., 1986). We were encouraged therefore to explore the possible use of this model for coprecipitation-transfection experiments to investigate the action of oncogenes on differentiated human epithelium. This has led us to the generation of differentiated immortal human thyroid epithelial cell lines which are highly suitable for further transfection experiments and are also likely to be useful for studies of the control of growth and function in the human thyroid.

### Materials and methods

### *Primary thyroid epithelial cell cultures*

These were prepared as we have previously described (Williams *et al*., 1987). Briefly, normal thyroid tissue was dissected from lobectomy specimens (surgically removed for solitary thyroid nodules) and digested with a mixture of collagenase (100 IU ml$^{-1}$) and dispase (1 mg ml$^{-1}$) and then the follicles were pooled, filtered through a 200 $\mu$m nylon mesh and washed with Hank's calcium- and magnesium-free balanced salt solution (HBSS). The follicular epithelium was plated as monolayers of $5 \times 10^5$ cells per 60 mm dish in RPMI 1640 medium with 10% fetal calf serum and 0.1 mU ml$^{-1}$ bTSH (bovine thyrotropic hormone; Sigma). Transfection was performed 4 days later. Thyroid follicular epithelium from one female patient aged 35 years was used for these cultures.

### *Transfection protocol*

Transfection was performed by the method of strontium phosphate coprecipitation (Brash *et al*., 1987). Six hours before transfection, the medium was replaced with 5 ml of warm SF-12 medium (Flow Labs) with 10% fetal calf serum. One $\mu$g of the plasmid SV40ori− (which comprises the 5.3 kb SV40 genome with a six base pair deletion that eliminates the BglI site at the origin of replication cloned in pMK16; Gluzman *et al*., 1980*a*, *b*) in 220 $\mu$l of water was mixed with 30 $\mu$l of 2 M strontium chloride and then added dropwise to 250 $\mu$l of 2 × Hepes-buffered saline. The resultant coprecipitate was left in contact with the epithelial monolayer for 90 min, and then the dishes were rinsed with serum-free medium before incubation with 1.5 ml of 15% glycerol in HBSS for 30 s at room temperature. After washing with HBSS, the cultures were refed with warm RPMI 1640 with 10% FCS + 0.1 mU ml$^{-1}$ bTSH.

### *Immunocytochemistry*

Dishes were fixed for 20 min in ice-cold 50% acetone/50% methanol and allowed to dry at room temperature. Expression of SV40 large T protein was detected by an indirect immunoperoxidase procedure using monoclonal antibody PAb419 (kindly supplied by Dr David Lane, ICRF London) as primary antibody followed by rabbit anti-mouse Ig conjugated to horseradish peroxidase (Miles Laboratories) as second antibody. Sites of antibody binding were visualised by the deposition of brown polymer following incubation in diaminobenzidine (DAB)/hydrogen peroxide (Graham & Karnovsky, 1966). Expression of human epithelial cytokeratins was confirmed by immunocytochemistry with the monoclonal antibody CAM 5.2 (Makin *et al*., 1984).

### *Iodide trapping assay*

This was performed on the immortalised cell lines at passage 20 and again at passage 80 by a method modified from that

Correspondence: N.R. Lemoine.
Received 3 February 1989; and in revised form 17 August 1989.

of Weiss et al. (1984). Each cell type was seeded at $2 \times 10^5$ cells per well of a 24-well plate in RPMI containing 10% serum and 0.1 mU $ml^{-1}$ bTSH and incubated for 2 days, after which they were washed and incubated in serum/bTSH-free medium for a further 2 days. Then the cultures were refed with serum-free RPMI containing various concentrations of bTSH and incubated for 4 days. Radioactive iodide ($Na^{125}I$, 0.2 µCi per well in 3.6 µM NaI solution) was administered to the cells and the incubation continued for 40 min. The medium was removed and the cells washed quickly with ice-cold Hank's balanced salt solution. One ml of 100 mM NaOH solution was then added to each well to solubilise the cells and the total iodide-125 in the cell lysate assessed with a gamma counter (four wells per point).

The protein-bound (organified) iodide-125 was precipitated using a final concentration of 40% (w/v) trichloroacetic acid and measured by gamma counter (four wells per point).

*Thyroglobulin assay*

Thyroglobulin production was measured at passage 20 using an enzyme-linked immunosorbent assay described by Kilduff et al. (1985).

*Southern blot analysis of integrated SV40 sequences*

This was performed on cultures at passage 40 as previously described (Lemoine et al., 1988) using nylon filters (Hybond, Amersham) and a vacuum blotting system (Vacugene, LKB Pharmacia). Genomic DNA from each cell line was digested with restriction enzymes BglI, SstI or XbaI (there is no restriction site for any of these enzymes in the transfected plasmid pSVori−) or with KpnI (for which there is a single restriction site in pSVori−). Transfected sequences were detected using the 5.3 kb BamHI insert of pSVori− $^{32}P$-labelled by the random primer method. Filters were washed under stringent conditions in 0.1 × SSC at 65 C (1 × SSC is 0.15 M NaCl, 0.015 M sodium citrate).

*Chromosome analysis*

Chromosomes were prepared by standard procedures (Watt & Stephens, 1976) and G-banded using 2 × SSC at 60°C for 15 min and Wrights stain. Fifty metaphases were counted from each cell line at passage 80 and at least 18 of these were fully analysed.

*Determination of anchorage requirement*

Cells were seeded in Methocel suspension, using a modification of a published method (Risser & Pollack, 1974). Sixty mm diameter culture dishes were coated with RPMI 1640 containing 0.9% agar, and duplicate dishes were inoculated with $10^2$, $10^3$ or $10^4$ cells suspended in RPMI 1640 containing 1.2% Methocel and 10% fetal calf serum. One ml of this Methocel medium was added each week, and after 4 weeks of total incubation the number of clones greater than 0.2 mm diameter was scored by microscopic examination of dishes fixed in 10% formalin and stained with 0.001% crystal violet. A diameter of 0.2 mm was chosen as the cut-off point because all the epithelial cells tested had a tendency to remain as persistent clumps on initial plating, but these clumps (which could potentially be scored falsely as positive) remained at less than 0.2 mm diameter. In parallel with these Methocel cultures, duplicate dishes containing $10^2$ cells in normal medium were seeded and, after 2 weeks culture, fixed and stained for counting of colonies ($10^2$ colonies could be conveniently counted, but larger seeding inocula caused difficulty in counting and so were not used on plastic). Normal human primary thyroid cells were used as negative controls and pEJ-transformed NIH3T3 cells as positive controls. The anchorage requirement of each cell line could then be expressed as the percentage of colonies in Methocel to colonies on plastic dishes.

*Tumorigenicity in nude mice*

Athymic (nude) mice were injected subcutaneously at 6 weeks old with $10^6$ cells suspended in 0.2 ml of growth medium. Animals were monitored for the appearance of tumours for 3 months from the time of injection. Half of the animals injected in each case were given 0.1% aminotriazole in their drinking water in order to raise the level of circulating TSH (Gibson & Doniach, 1967). Control experiments included injections of $10^6$ untransfected normal primary human thyroid epithelial cells (which produced no tumours) and injections of $10^6$ NIH 3T3 fibroblasts transformed by an activated Ha-*ras* oncogene (which regularly produced tumours within 7–8 days).

*Determination of growth factor requirement of cell lines*

Replicate 35 mm dishes were seeded with $10^4$ cells in RPMI 1640 medium supplemented with 10% calf serum and 0.1 mU $ml^{-1}$ bTSH, and after 16 h medium was changed to RPMI containing either no growth factors or fetal calf serum at 1–10%. After 4 days of incubation, proliferative activity was determined by $^3H$-thymidine labelling of replicate dishes during a 1 h 'window': $^3H$-thymidine was added to a final concentration of 20 µCi $ml^{-1}$, then 1 h later the dishes were fixed in methanol/acetic acid (3:1), coated in Ilford K2 emulsion and exposed in the dark (4°C, 6 days), developed and counterstained with Giemsa (Williams et al., 1987). For each data point, the nuclear labelling index in a random count of 1,000 nuclei was scored.

*Determination of transfection efficiency of cell lines*

Transfection was carried out using the protocol of strontium phosphate coprecipitation detailed above, with $5 \times 10^5$ cells in 60 mm dishes transfected with 1–10 µg of pSV2neo. Twenty-four hours after the coprecipitate was washed off, the cultures were passed into 140 mm dishes in medium containing 400 µg $ml^{-1}$ G418. Cultures were refed with fresh selective medium twice weekly and G418-resistant colonies scored in fixed and stained dishes (five dishes at each dose of plasmid) after 2 weeks.

### Results

*In vitro establishment of human thyroid epithelium by SV40 DNA transfection*

Normal human primary thyroid follicular epithelium undergoes only a few divisions before senescence of the culture. It was therefore relatively easy to monitor the establishment of transfected clones with an extended lifespan, which appeared as actively dividing colonies of rounded cells against the background of the degenerating senescent culture at about 3–4 weeks. The frequency of these transformants was usually about 1–2 per $10^4$ cells transfected, and immunocytochemistry of fixed cultures confirmed that SV40 large T antigen was expressed in these colonies. Only those colonies that had an obviously epithelial phenotype (closely packed cuboidal cells growing in discrete islands) were picked, when they comprised about 2,000 cells, and grown to confluence in 35 mm dishes. Thereafter, the clones were passaged at 1:4 ratio every 3–4 days. All 10 clones picked have been through at least 40, and HTori-3 and HTori-5 have now been through over 100 passages without overt crisis.

*Iodide trapping and thyroglobulin secretion*

Ten clones were subjected to these analyses in order to isolate a cell line that retained differentiated characteristics. Two clones (HTori-3 and HTori-5) showed good evidence of specific thyrocyte function when tested after 20 passages, being able to trap iodide actively, and in the case of HTori-5 showing a similar dose-response to TSH to that of normal

**Figure 1** Effect of bovine TSH concentration on iodide-trapping activity of human thyroid follicular cells and cell lines. Each data point represents the mean ± s.e. (four replicate experiments).

primary epithelium (Figure 1). Retesting of the lines at passage 80 confirmed retention of active iodide-trapping activity, although the marked TSH-induced stimulation observed in early passages of HTori-5 was no longer seen (Figure 1). Control cultures of human primary fibroblasts showed no detectable iodide-trapping activity in this assay. Organification of the trapped iodide did not occur in any of the immortal cell lines.

Assay of thyroglobulin production also confirmed the specific function of these two lines: while normal primary thyroid cultures produced 790 ng thyroglobulin per ml of medium per $10^5$ cells, HTori-3 produced 79 ng ml$^{-1}$ per $10^5$ cells and HTori5 produced 75 ng ml$^{-1}$ per $10^5$ cells (human primary fibroblasts produced no detectable thyroglobulin). Accordingly, these two clones (HTori-3 and HTori-5) were chosen for more detailed characterisation.

*Morphological characteristics*

Both HTori-3 and HTori-5 are clearly epithelial in morphology, with a tendency to grow as closely packed islands of cells; they are more refractile and mitotically active than the normal primary cells from which they were derived (Figure 2). Their epithelial nature is supported most strongly by the specific functional responses described above, but also by their cytoplasmic immunoreactivity for epithelial keratin (Figure 2).

*SV40 DNA integration*

Southern blot analysis showed the presence of SV40 DNA in the genomes of both thyroid cell lines. With the SVori− non-cutters (BglI, SstI, XbaI) both cell lines (HTori-3 and HTori-5) show single major integration sites for the transfected plasmid (Figure 3). With KpnI, two fragments are seen in each cell line DNA (different in each case). These data indicate that the two cell lines are independent, and each is the result of integration of a single copy of SVori−.

*Chromosome analysis*

Both HTori-3 and HTori-5 were aneuploid with many complex chromosome aberrations. HTori-3 had a range of 44–76 chromosomes per metaphase, with no clear mode. One to six double minutes were observed in 12% of cells analysed. Several marker chromosomes were noted in over 20% of cells analysed, including 1q+ (M3 in Figure 4) in 77% of cells and 11q− (M18 in Figure 4) in 45% of cells. HTori-5 had a range of 51–80 chromosomes per metaphase, with no clear mode. One to two double minutes were seen in 10% of cells analysed. Markers seen in at least 20% of cells included 1q+ (M2 in Figure 4) in 20% of cells and 2p+ (M3 in Figure 4) in 28% of cells.

**Figure 3** Southern blot analysis of integrated SV40 sequences in HTori-3 (lanes **b**–**e**) and HTori-5 (lanes **f**–**i**) cell lines. Ten micrograms of each DNA digested with BglII (lanes **b** and **f**), SstI (lanes **c** and **g**), XbaI (lanes **d** and **h**) or KpnI (lanes **e** and **i**). The control lane (**a**) contains pSVori− plasmid DNA digested with KpnI. The presence of a single band after digestion with each insert non-cutter (BglII, SstI, XbaI) and only two bands after digestion with KpnI (which cuts the insert of Svori− once) indicates a single integrated copy of SVori−. Size markers in kbp.

**Figure 2** Appearance of monolayers of normal primary human thyroid epithelium (**a**) and the SV40-immortalised human thyroid cell line HTori-3 (**b**). The SV40-transformed cells are more rounded and refractile than the normal primary cells and there are many mitotic cells within the confluent monolayer. Immunoreactivity with monoclonal antibodies against SV40 large T antigen (**c**) and epithelial cytokeratins (**d**), showing nuclear and cytoplasmic immunoreactivity respectively, in monolayers of HTori-3 cell line. HTori-5 showed similar immunoreactivities.

**Figure 4** Karyotype of (**a**) HTori-3 and (**b**) HTori-5 with marker chromosomes ranked at lower part of figure.

*Anchorage independence and tumorigenicity*

Both HTori-3 and HTori-5 have a low but significant ability to form anchorage-independent colonies in Methocel which has remained stable over multiple passages. HTori-3 forms anchorage-independent colonies at 0.3% efficiency and HTori-5 at 0.4% efficiency, compared to 74% efficiency for pEJ-transformed NIH3T3 cells. Neither line is capable of forming tumours in nude mice, even in the presence of elevated TSH (animals treated with aminotriazole).

*Growth factor dependence*

Both cell lines are capable of DNA synthesis in 0% serum (as measured by nuclear labelling following thymidine incorporation) in contrast to normal primary thyroid epithelial cultures which become quiescent under these conditions (Figure 5). Although they can both sustain proliferation independent of the presence of serum, both lines are capable of dramatic increase of proliferative index in response to challenge with 10% serum (Figure 5). A specific proliferative response to TSH could not be demonstrated either in the presence or absence of serum.

*Transfection efficiency of SV40-transformed cell lines*

Both lines could be efficiently and stably transformed to G418 resistance by strontium phosphate transfection of the *neo* gene (Figure 6), HTori-3 showing slightly higher frequencies at all doses of transfected plasmid. Under optimum conditions, the transfection frequency was at least 2 per 1,000 cells transfected. Approximately 15 μg of plasmid was required to saturate the transfection capacity of each line (Figure 6).

**Figure 5** $^{3}$H-thymidine labelling index of human thyroid follicular cells and cell lines in response to various concentrations of fetal calf serum.

**Figure 6** Graph showing transfectability of the two human thyroid cell lines HTori-3 and HTori-5, using test system of conversion to G418-resistance following transfection of pSV2neo plasmid by strontium phosphate coprecipitation. Each data point represents the mean ± s.e. of five transfected dishes.

### Discussion

The human thyroid follicular cell has several attractive features as a model of epithelial tumorigenesis. In terms of cell kinetics and differentiation, the thyroid gland can be regarded as a homogeneous cell population controlled *in vivo* by a single major specific mitogen, thyroid-stimulating hormone (Redmond & Tuffery, 1981; Wynford-Thomas *et al.*, 1982). *In vitro*, human follicular cells can be easily cultured in chemically defined, serum-free media, allowing extensive dissection of its growth control mechanisms. To these advantages can now be added the suitability of these cells for gene transfer experiments.

The purpose of these experiments was to generate immortal human thyroid follicular cell lines that would be suitable for further genetic manipulation; two such lines, which retain thyroid-specific differentiated features, have been established and shown to be suitable for introduction of cloned genes by transfection.

These experiments have shown that human thyroid epithelial cells can be immortalised without crisis and partially transformed after transfection of SV40 DNA. The resultant immortal cell lines retain an epithelial morphology and (in some cases) retain features of specific differentiation, but do not require the presence of serum for proliferation and have a significant capacity for anchorage-independent growth. None of the cell lines was capable of forming tumours in nude mice.

These results are in broad agreement with those obtained for SV40-mediated transformation of other human epithelial cell types: immortalisation, conversion to anchorage-independent and relative independence of exogenous growth factors are common features of these experiments. The level of anchorage independence observed in our lines (0.2%, 0.4%) is similar to that seen in other SV40-transformed human epithelial lines, such as colonic cells (0.3%; Berry *et al.*, 1988) and urothelial cells (0.03–3.0%; Christian *et al.*, 1987). Acquisition of tumorigenicity in nude mice after introduction of SV40 genes alone has rarely been observed in human epithelial cells, or indeed in other human cell types (Kahn *et al.*, 1983). It is not clear why SV40-transformed *human* epithelial cells are usually non-tumorigenic in nude mice (an exception being late-passage SV40-transformed keratinocytes; described by Brown & Gallimore, 1987), while SV40-transformed *rodent* epithelial cells, such as rat hepatocytes (Woodworth *et al.*, 1988), are often strongly tumorigenic (Freedman & Shin, 1978). The fact that some SV40-transformed human epithelial cell lines, such as the 184A1 mammary line (Clark *et al.*, 1988) and the HBL-100 mammary line (Saint-Ruf *et al.*, 1988), can be converted to tumorigenicity by *ras* oncogenes (activated Ha-*ras* and Ki-*ras* respectively) strongly suggests that the parent lines are indeed incompletely transformed.

Both of the thyroid cell lines (HTori-3 and HTori-5) subjected to chromosome analysis were aneuploid, which is to be expected after transformation by SV40. Periodic re-analysis in the future will be required to determine the stability of the genomes, but evidence from most other studies suggests that aberrations are likely to increase with progressive generations (Meisner *et al.*, 1988; Brown & Gallimore, 1987). The apparent integration of only a single copy of SV40 plasmid in each of the established cells lines analysed corresponds with previous observations in other human cell types (Neufeld *et al.*, 1987; Canaani *et al.*, 1986; Murnane *et al.*, 1985) and it seems that immortalisation is also favoured by a low copy number of integrated SV40 DNA in cells infected with wild-type SV40 (Kucherlapati *et al.*, 1978; Hara & Kaji, 1987).

Partial retention of differentiated phenotype in two of the immortalised thyroid cell lines could make them particularly useful for future studies of the relationship between differentiation and transformation. The reduced level of iodide-trapping activity after extended passage of these lines is similar to that which we have observed in SV40-transformed rat thyroid cells (Burns *et al.*, 1989). Other human epithelial cell types transformed by SV40 show either a partial loss (such as keratinocytes; Banks-Schlegel & Howley, 1983) or complete loss (such as urothelial cells; Christian et al., 1987) of their normal differentiated phenotype. The two differentiated lines, HTori-3 and HTori-5, are likely to be useful also to investigators of the basic mechanisms of human thyroid function, as well as those concerned with thyroid neoplasia, since cell lines capable of thyroglobulin production have not previously been available. Both the SGHTL-34 (Whitley et al., 1987) and 12S (Cone et al., 1988) human thyroid lines have been shown to possess a specific TSH-sensitive adenylate cyclase response (although much reduced compared to primary thyroid follicular cells), but do not apparently produce thyroglobulin. The only other human thyroid follicular cells lines are products of cell fusion between thyroid follicular cells and human lymphoid lines (GEJ line of Karsenty et al. (1985); HY2-15 line of Martin et al. (1988)); the GEJ line has proved to be very unstable, while the HY2-15 line retains only adenylate cyclase response to TSH. Animal cell lines are available, such as the rat FRTL-5 line (Ambesi-Impiombato et al., 1980) and the ovine OVNIS-5H line (Fayet et al., 1986), but have the disadvantage of species specificity.

The transfectability of HTori-3 and HTori-5 makes them very suitable for neoplastic reconstruction experiments involving the introduction of cloned oncogenes. The peak frequency of transformation to G418 resistance (1 per 1,000 cells transfected) is of the same order as the NIH3T3 fibroblast line, the NMuMG mouse mammary cell line (Hynes et al., 1985), and the HOS human osteosarcoma cell line (Tainsky et al., 1987) all of which have been proposed as acceptor cells for the transfection of genomic DNA. However, these latter cell lines show higher sensitivity to low copy number genes (giving one to five G418-resistant colonies per ng pSV2neo plasmid), and maximum yield of transformants is obtained with approximately 1 µg plasmid per 60 mm dish compared to 15 µg per 60 mm dish for HTori-3 and HTori-5. The latter result indicates that although the proportion of transfectable cells in a population of HTori-3 or HTori-5 is comparable to that in NIH3T3, the *amount* of DNA taken up by each transfectable cell is much lower. This is in agreement with a previous study which showed that the average amount of DNA stably incorporated by human (fibroblast) cells was 20- to 100-fold lower than that incorporated by rodent (Chinese hamster fibroblast) cells (Hoeijmamakers et al., 1987). The conclusion from our experiments must be that, in common with most other human cells, the human thyroid follicular cell lines HTori-3 and HTori-5 are eminently suitable for transfection studies using *cloned* genes, but at present unsuitable for transfection with *genomic* DNA.

The model system which we have established here will allow us to explore the action of other oncogenes alone and in combination with SV40 sequences. We have shown that activated *ras* oncogenes are frequently present in both benign and malignant human thyroid tumours (Lemoine et al., 1988, 1989) and so these oncogenes will be the first priority for analysis. The work of Ridley et al. (1988) suggests that SV40 sequences may be particularly useful as a complementing agent in investigating the transforming action of *ras* oncogenes, which alone may have an inhibitory effect on cell proliferation.

Preliminary experiments show that these cell lines are also susceptible to efficient transformation by amphotrophic retroviral vectors (data not shown), which further increases the flexibility of this model system.

This work was supported by a grant from the Welsh Scheme for the Development of Health Service Resources.

### References

AMBESI-IMPIOMBATO, F.S., PARKS, L.A.M. & COON, H.G. (1980). Culture of hormone-dependent functional epithelial cells from rat thyroids. *Pro. Natl Acad. Sci. USA*, **77**, 3455.

BANKS-SCHLEGEL, S.P. & HOWLEY, P.M. (1983). Differentiation of human epidermal cells transformed by SV40. *J. Cell Biol.*, **96**, 330.

BERRY, R.D., POWELL, S.C. & PARASKEVA, C. (1988). *In vitro* culture of human foetal colonic epithelial cells and their tranformation with origin minus SV40 DNA. *Br. J. Cancer*, **57**, 287.

BOUKAMP, P., STANBRIDGE, E.J., CERUTTI, P.A. & FUSENIG, N.E. (1986). Malignant transformation of 2 human skin keratinocyte cell lines by Harvey-*ras* oncogene (abstract). *J. Invest. Dermatol.*, **87**, 131.

BRASH, D.E., REDDEL, R.R., QUANRUD, M., YANG, K., FARRELL, M.P. & HARRIS, C.C. (1987). Strontium phosphate transfection of human cells in primary culture: stable expression of the simian virus 40 large-T-antigen gene in primary human bronchial epithelian cells. *Mol. Cell. Biol.*, **7**, 2031.

BROWN, K.W. & GALLIMORE, P.H. (1987). Malignant progression of an SV40-transformed human epidermal keratinocyte cell line. *Br. J. Cancer*, **56**, 545.

BURNS, J.S., LEMOINE, L., LEMOINE, N.R., WILLIAMS, E.D. & WYNFORD-THOMAS, D. (1989). Thyroid epithelial cell transformation by a retroviral vector expressing SV40 large T. *Br. J. Cancer*, **59**, 755.

CANAANI, D., NAIMAN, T., TEITZ, T. & BERG, P. (1986). Immortalization of Xeroderma Pigmentosum cells by simian virus 40 DNA having a defective origin of DNA replication. *Somat. Cell Mol. Genet.*, **12**, 13.

CHANG, S.E., KEEN, J., LANE, E.B. & TAYLOR-PAPADITROU, J. (1982). Establishment and characterisation of SV40-transformed human breast epithelial cell lines. *Cancer Res.*, **42**, 2040.

CHANG, S.E. (1986). *In vitro* transformation of human epithelial cells. *Biochim. Biophys. Acta*, **823**, 161.

CHRISTIAN, B.J., LORETZ, L.J., OBERLEY, T.D. & REZNIKOFF, C.A. (1987). Characterization of human uroepithelial cells immortalized *in vitro* by simian virus 40. *Cancer Res.*, **47**, 6066.

CLARK, R., STAMPFER, M.R., MILLER, R. & 5 others (1988). Transformation of human mammary epithelial cells by oncogenic retroviruses. *Cancer Res.*, **48**, 4689.

CONE, R.D., PLATZER, M., PICCININI, L.A., JARAMILLO, M. & DAVIES, T.F. (1988). HLA-DR gene expression in a proliferating human thyroid cell clone (12S). *Endocrinology*, **123**, 2067.

DIPAOLO, J.A. (1983). Relative difficulties in transforming human and animal cells *in vitro*. *J. Natl Cancer Inst.*, **70**, 3.

FAYET, G., AOUANI, A. & HOVSEPIAN, S. (1986). TSH-induced cyclic AMP production in an ovine thyroid cell line: OVNIS 5H. *FEBS Lett.* **194**, 287.

FREEDMAN, V.H. & SHIN, S. (1978). Use of nude mice for studies on the tumourigenicity of animal cells. In *The Nude Mouse in Experimental and Clinical Research*, Fogh & Giovanella (eds) p. 353. Academic Press: New York.

GIBSON, J.M. & DONIACH, I. (1967). Correlation of dose of X-radiation to the rat thyroid gland with degree of subsequent impairment of response to goitrogenic stimulus. *Br. J. Cancer*, **21**, 524.

GIRARDI, R.J., JENSEN, F.C. & KOPROWSKI, H. (1965). SV40-induced transformation of human diploid cells: crisis and recovery. *J. Cell. Comp. Physiol.*, **65**, 69.

GLUZMAN, Y., FRISQUE, R.J. & SAMBROOK, J. (1980*a*). Origin-defective mutants of SV40. *Cold Spring Harbor Symp. Quant. Biol.*, **44**, 293.

GLUZMAN, Y., SAMBROOK, J. & FRISQUE, R.J. (1980*b*). Expression of early genes of origin-defective mutants of simian virus 40. *Proc. Natl Acad. Sci. USA*, **77**, 3898.

GRAHAM, R.C. & KARNOVSKY, M.J. (1966). The early stages of absorption of injected horseradish peroxidase in the proximal tubules of mouse kidney: ultrastructural cytochemistry by a new technique. *Histochem. Cytochem.*, **14**, 291.

HARA, H. & KAJI, H. (1987). Random integration of SV40 in SV40-transformed, immortalized human fibroblasts. *Exp. Cell Res.*, **168**, 531.

HOEIJMAKERS, J.H.J., ODIJK, H. & WESTERVELD, A. (1987). Differences between rodent and animal cell lines in the amount of integrated DNA after transfection. *Exp. Cell. Res.*, **169**, 111.

HYNES, N.E., JAGGI, R., KOZMA, S.C. & 5 others (1985). New acceptor cell for transfected genomic DNA: oncogene transfer into a mouse mammary epithelial cell line. *Mol. Cell. Biol.*, **5**, 268.

KAHN, P., TOPP, W.C. & SHIN, S.I. (1983). Tumorigenicity of SV40-transformed human and monkey cells in immunodeficient mice. *Virology*, **126**, 348.

KARSENTY, G., MICHEL-BECHET, M. & CHARRIERE, J. (1985). Monoclonal human thyroid cell line GEJ expressing human thyrotropin receptors. *Proc. Natl Acad. Sci. USA*, **82**, 2120.

KILDUFF, P., BLACK, E.G., HALL, R. & McGREGOR, A.M. (1985). An enzyme-linked immunosorbent assay for the measurement of thyroglobulin in human serum using monoclonal antibodies. *J. Endocrinol.*, **107**, 383.

KUCHERLAPATI, R., HWANG, S.P., SHIMUZU, N., McDOUGALL, J.K. & BOTCHAN, M.R. (1978). Another chromosomal assignment for a simian virus 40 integration site in human cells. *Proc. Natl Acad. Sci. USA*, **75**, 4460.

LEMOINE, N.R., MAYALL, E.S., WILLIAMS, E.D., THURSTON, V. & WYNFORD-THOMAS, D. (1988). Agent-specific ras oncogene activation in rat thyroid tumours. *Oncogene*, **3**, 541.

LEMOINE, N.R., MAYALL, E.S., WYLLIE, F.S. & 4 others (1989). High frequency of ras oncogene activation in all stages of human thyroid tumorigenesis. *Oncogene*, **4**, 159.

MAKIN, C.A., BOBROW, L.G. & BODMER, W.F. (1984). Monoclonal antibody to cytokeratin for use in routine histopathology. *J. Clin. Pathol.*, **37**, 975.

MARTIN, A., PLATZER, M. & DAVIES, T.F. (1988). Retention of cyclic AMP response to TSH in a cloned human thyrocyte/T cell hybridoma (HY2-15). *Mol. Cell. Endocrinol.*, **60**, 233.

MEISNER, L.F., WU, S., CHRISTIAN, B.J. & REZNIKOFF, C.A. (1988). Cytogenetic instability with balanced chromosome changes in an SV40 transformed human uroepithelial cell line. *Cancer Res.*, **48**, 3215.

MURNANE, J.P., FULLER, L.F. & PAINTER, R.B. (1985). Establishment and characterization of a permanent pSVori- transformed Ataxia Telangiectasia cell line. *Exp. Cell Res.*, **158**, 119.

NEUFELD, D.S., RIPLEY, S., HENDERSON, A. & OZER, H.L. (1987). Immortalization of human fibroblasts transformed by origin-defective simian virus 40. *Mol. Cell. Biol.*, **7**, 2794.

PARKIN, D.M.E. & MUIR, C.S. (1988). Estimates of the worldwide frequency of sixteen major cancers in 1980. *Int. J. Cancer*, **41**, 184.

REDDEL, R.R., KE, Y., GERWIN, B.I. & 7 others (1988). Transformation of human bronchial epithelial cells by infection with SV40 or adenovirus-12 SV40 hybrid virus, or transfection via strontium phosphate coprecipitation with a plasmid containing SV40 early region genes. *Cancer Res.*, **48**, 1904.

REDMOND, O. & TUFFERY, A.R. (1981). Mitotic rate of rat thyroid follicular cells *in vivo* in response to a single injection of thyrotropin (TSH). *Cell Tissue Kinet.*, **14**, 625.

RIDLEY, A.J., PATERSON, H.F., NOBLE, M. & LAND, H. (1988). *ras*-mediated cell cycle arrest is altered by nuclear oncogenes to induce Schwann cell transformation. *EMBO J.*, **7**, 1635.

RISSER, R. & POLLACK, R. (1974). Nonselective analysis of SV40 transformation of mouse 3T3 cells. *Virology*, **59**, 471.

SAGER, R. (1984). Resistance of human cells to oncogenic transformation. In *Cancer Cells 2: Oncogenes and Viral Genes*, p. 487. Cold Spring Harbor Press.

SAINT-RUF, C., NARDEUX, P., ESTRADE, S. & 4 others (1988). Accelerated malignant progression of human HBL-100 cells by the v-Ki-*ras* oncogene. *Exp. Cell Res.*, **176**, 60.

TAINSKY, M.A., SHAMANSKI, F.L., BLAIR, D. & VANDE WOUDE, G. (1987). Human recipient cell for oncogene transfection studies. *Mol. Cell. Biol.*, **7**, 1280.

TAYLOR-PAPADIMITROU, J., PURKIS, P., LANE, E.B., McKAY, I.A. & CHANG, S.E. (1982). Effects of SV40 transformation on the cytoskeleton and behavioural properties of human keratinocytes. *Cell Different.*, **11**, 169.

TUR-KASPA, R., TEICHER, L., LEVINE, B.J., SKOULTCHI, A.I. & SHAFRITZ, D.A. (1986). Use of eletroporation to introduce biologically active foreign genes into primary rat hepatocytes. *Mol. Cell. Biol.*, **6**, 716.

WATT, J.L., & STEPHENS, G.S. (1986). Lymphocyte culture for chromosome analysis. In *Human Cytogenetics: a Practical Approach*, Rooney, D.E. & Czepulowski, B.H. (eds) p. 39. IRL Press: Oxford.

WEISS, S.J., PHILP, N.J. & GROLLMAN, E.F. (1984). Iodide transport in a continuous line of cultured cells from rat thyroid. *Endocrinology*, **114**, 1090.

WHITLEY, G.ST.J., NUSSEY, S.S. & JOHNSTONE, A.P. (1987). SGHT1-34, a thyrotrophin-responsive immortalised human thyroid cell line generated by transfection. *Mol. Cell. Endocrinol.*, **52**, 279.

WILLIAMS, D.W., WYNFORD-THOMAS, D. & WILLIAMS, E.D. (1987). Control of human thyroid follicular cell proliferation in suspension and monolayer culture. *Mol. Cell. Endocrinol.*, **51**, 33.

WOODWORTH, C.D., KREIDER, J.W., MENGEL, L., MILLER, T., MENG, Y. & ISOM, H.C. (1988). Tumorigenicity of simian virus 40-hepatocyte cell lines: effect of *in vitro* and *in vivo* passage on expression of liver-specific genes and oncogenes. *Mol. Cell. Biol.*, **8**, 4492.

WYNFORD-THOMAS, D., STRINGER, B.M.J. & WILLIAMS, E.D. (1982). Dissociation of growth and function in the rat thyroid during prolonged goitrogen administration. *Acta Endocrinol.*, **101**, 210.

YOAKUM, G.H., LECHNER, J.F., GABRIELSON, E. & 7 others (1985). Transformation of human bronchial epithelial cells transfected by Harvey *ras* oncogene. *Science*, **227**, 1174.